# Help-seeking behaviour in dysmenorrhoea: A cross-sectional exploration using the Behavioural Model of Health Services Use

**DOI:** 10.1177/17455057241273588

**Published:** 2024-08-20

**Authors:** Sophie C Matheson, Hannah Durand

**Affiliations:** Division of Psychology, University of Stirling, Stirling, Scotland, UK

**Keywords:** menstruation, dysmenorrhoea, menstrual pain, help-seeking, pain interference

## Abstract

**Background:**

Dysmenorrhoea, or period pain, is a prevalent gynaecological condition that can result in functional interference during menstruation. Despite the significant disruption dysmenorrhoea can have on functioning and well-being, medical help-seeking rates are low. Little is known about what factors may predict help-seeking for dysmenorrhoea.

**Objectives:**

The current study aimed to test the predictive validity of the Behavioural Model of Health Services Use (BMHSU) for help-seeking behaviour in dysmenorrhoea, whereby help-seeking behaviour was operationalised as having attended to a healthcare professional for dysmenorrhoea-related care.

**Design:**

A cross-sectional observational design was used.

**Methods:**

Participants (*N* = 439) completed an online survey, which measured the following eight predictor variables: menstrual pain characteristics, health beliefs, self-efficacy, social support utilisation and satisfaction, perceived healthcare availability, and pain intensity and interference. Participants were also asked to report whether they had ever attended to a healthcare professional for their menstrual pain.

**Results:**

The BMHSU accounted for 8% of the variance in help-seeking behaviour. Pain interference and appointment availability were significant predictors of the variance in past help-seeking behaviour, such that those who experienced greater pain interference, and those who perceived greater availability of healthcare appointments were less likely to have visited a healthcare professional for their menstrual pain. The BMHSU had an overall 69% classification accuracy in predicting help-seeking behaviour.

**Conclusion:**

Although the BMHSU demonstrated reasonably good model fit, it does not appear to be a particularly robust model for predicting help-seeking behaviour for dysmenorrhoea. Future research should explore whether a refined BMHSU or an alternative theoretical model can provide more useful insight into this behaviour. Better understanding of the determinants of help-seeking behaviour will enable the development of interventions to promote appropriate help-seeking and improve health outcomes for individuals with menstrual pain.

## Plain language summary

What makes people with period pain seek help?

Many people experience common period pain, which can be severe and debilitating. Even though it can make daily life difficult, not many people seek medical help for period pain.

The purpose of this study was to find out why people with period pain might or might not go to a doctor for help. We used a theoretical framework called the Behavioural Model of Health Services Use to try to understand this. This framework tells us that predisposing factors like age or health beliefs, enabling factors like family support and access to healthcare, and need factors like severe symptoms, can predict whether someone will go to the doctor. We wanted to test if this way of thinking could accurately predict if someone with period pain had been to see a doctor.

We asked people with period pain to fill in an online survey. We asked about things like how painful their periods were, what they believed about their menstrual health, how confident they felt in managing their pain, whether they got support from others, if they thought healthcare was available, and how much the pain affected their lives. We also asked if they had ever gone to a doctor for help with their period pain.

We found that these factors were not very accurate in predicting whether people sought help for period pain. The most important predictors were how much the pain affected their lives and whether they believed that healthcare appointments were available to them. If the pain was very disruptive, and if they felt there were free appointments, people were less likely to have gone to see a doctor.

This study was the first to use this way of thinking to understand help-seeking for people with period pain. In the future, researchers should test different models to see if they work better for understanding help-seeking behaviour for period pain. It is important to find ways to understand this behaviour to help people seek help for their pain when they need it.

## Introduction

Dysmenorrhoea (i.e. pain of uterine origin experienced just before or during menstruation) is a prevalent gynaecological condition.^1^ It can occur secondary to gynaecological disorders such as endometriosis, adenomyosis, fibroids, or pelvic inflammatory disorder, or as a primary form of disease.^2^ Symptoms of dysmenorrhoea can include pain in the lower abdomen, back and legs, diarrhoea, nausea, and vomiting.^3^ It is estimated that between 45% and 95% of all individuals who menstruate globally experience dysmenorrhoea.^4^ In individuals with dysmenorrhoea, the pain and associated symptoms can be debilitating.^5^ A recent systematic review and meta-analysis found that symptoms of dysmenorrhoea negatively impacted concentration and classroom performance in 40.9% of young women.^6^ Furthermore, 20.1% reported their menstrual pain had caused them to be absent from school or university. Dysmenorrhoea can also significantly reduce individuals’ quality-of-life. Iacovides and colleagues^7^ found that women with dysmenorrhoea had significantly lower quality-of-life scores compared to pair-matched controls. Additionally, those with dysmenorrhoea also reported significantly lower quality-of-life when they were menstruating compared to when they were not menstruating. Despite its prevalence and impact, dysmenorrhoea remains poorly understood and under-researched.^8^

Although dysmenorrhoea can have substantial negative effects on a person’s life,^2^ most do not seek help from a medical professional.^9^ In a survey of 1266 female university students, the prevalence of dysmenorrhoea was 88%^10^; of those, only 19.1% consulted with a doctor. In a qualitative study with 225 women with dysmenorrhoea,^11^ findings indicated that people did not seek medical help or advice due to beliefs that medical professionals would not offer help, feelings of embarrassment about reaching out, not being aware of what types of treatment were available, and believing that dysmenorrhoea was normal. Some also stated that when they did seek help, the doctor did not believe their symptoms required treatment. This is consistent with quantitative research by Armour and colleagues,^12^ which found that 83.8% of 4202 females surveyed believed that dysmenorrhoea was normal. These beliefs have important implications for help-seeking behaviour. There has, however, been limited investigation of other potential predictors of help-seeking behaviour for individuals with dysmenorrhoea to date.

### Behavioural Model of Health Services Use

The Andersen Healthcare Utilisation Model is a theoretical framework based on a national quantitative survey to understand the utilisation of health and care services by families.^13^ Since its initial development, the model has undergone several adaptations, with the most comprehensive and frequently used version being the Behavioural Model of Health Services Use (BMHSU).^14,15^ The BMHSU comprises factors that facilitate or impede individuals’ use of health services, such as seeking help from a healthcare professional. This model can be applied to investigate barriers and facilitators of healthcare utilisation for individuals with specific diseases or conditions and may constitute a useful framework for understanding help-seeking for dysmenorrhoea.

The BMHSU conceptual framework consists of three categories that predict whether someone will engage in help-seeking behaviour or not. These categories are predisposing, enabling, and need factors. The BMHSU posits that the decision to visit a healthcare professional is influenced by the interaction of factors within these three categories. Predisposing factors are individual characteristics that affect the likelihood of utilising healthcare services, such as age, health beliefs, and self-efficacy. According to the Health Belief Model (HBM), health beliefs can predict health behaviours, and in turn, health outcomes.^16^ Positive health beliefs can include believing that you are in good health or believing that a medical treatment will be efficacious at improving health, whereas negative health beliefs include being pessimistic about your health or doubting that a medical treatment will improve well-being. Enabling factors are the environmental variables that facilitate or hinder access to healthcare services, such as country of residence, social support system utilisation and satisfaction, and perceived availability of health services. Need factors are variables related to an individual’s health status that may influence healthcare utilisation, such as pain intensity and pain interference. Predisposing, enabling, and need factors as conceptualised by the BMHSU have been demonstrated to be predictive of healthcare utilisation for a variety of health conditions, including painful conditions.^17^

The BMHSU has not been applied to help-seeking for dysmenorrhoea. There is, however, some evidence to suggest that the model may be applicable to this group. Predisposing factors of age and beliefs have been demonstrated to predict help-seeking behaviour for dysmenorrhoea and other pain conditions.^11,18^ Enabling factors like financial cost and social support have also been associated with help-seeking for dysmenorrhoea, specifically.^11,19,20^ Need factors like pain severity and pain-related disability and interference have also been identified as potential mediators for pain-related help-seeking.^18,21–23^ However, little research to date has used robust theoretical frameworks to guide investigations of help-seeking behaviours in dysmenorrhoea. Using established theory to help understand the ways in which these factors may affect help-seeking behaviour for dysmenorrhoea can increase our knowledge and, most importantly, enable development of strategies to target modifiable determinants to promote appropriate help-seeking for those whose pain interferes with daily functioning and psychosocial wellbeing.

### The current study

The BMHSU has been used extensively to understand barriers and facilitators of help-seeking behaviour for a range of health conditions.^17,24,25^ However, it has not been applied to help-seeking behaviour for dysmenorrhoea to date. The current study aimed to evaluate the predictive validity of the BMSHU for help-seeking behaviour amongst individuals with dysmenorrhoea living in the United Kingdom (UK).

## Methods

### Design

A cross-sectional (observational) quantitative online survey design was used. The Strengthening of the Reporting of Observational Studies in Epidemiology (STROBE) Guidelines^26^ were followed when preparing the manuscript (see Supplemental material File 1 for STROBE Checklist).

### Participants

Individuals in the UK who menstruate, regardless of gender identity, were invited to self-select into this study. Inclusion criteria were currently residing in the UK; being aged 16 years or older if resident in Scotland and 18 years or older if resident in England, Wales, or Northern Ireland; having reached menarche (i.e., had had their first menstrual period); and being premenopausal (i.e., had not reached perimenopause or menopause). In Scotland, individuals aged 16 years and over are presumed to be capable of giving consent on their own behalf to participate in research.^27^ Therefore, parental consent was not obtained. Participation was limited to those living in the UK in order to focus on one healthcare context. People living in the UK are assumed to have access to free healthcare via the National Health Service (NHS). A target sample size of *N* = 114 was determined using Green’s^28^ heuristic method (*n* > 50 + 8m, where m represents the number of predictor variables).

### Measures

Participants self-reported demographic data as well as information regarding their menstrual cycle, including whether they experienced menstrual pain. If they experienced pain, participants were further asked whether they had any previous diagnoses of underlying gynaecological conditions. Participants were asked to indicate which sources they used for guidance regarding their menstrual pain and which kind(s) of healthcare professional (if any) they had visited regarding their menstrual pain. A list was presented, and they were asked to select all answers that were relevant to them. Included within the list was an ‘other’ option where participants could type a response in a free-text box to give an answer not already included. Satisfaction with the healthcare they received was rated on a 10-point Likert scale ranging from 1 ‘not satisfied at all’ to 10 ‘totally satisfied’. Participants were also asked to complete the following measures:

#### Health beliefs

The Brief Survey of Pain Attitudes (SOPA-B)^29^ was used to measure health beliefs. The SOPA-B is a 30-item measure that assesses seven facets of pain attitudes (control, solicitude, medication, disability, emotionality, cure, and harm). Participants indicated how true the statements were to them on a five-point Likert scale from 0 ‘very untrue’ to 4 ‘very true’. After accounting for reverse-coded questions, the total score was determined by averaging the responses to the 30 statements. A higher average score indicated more negative health beliefs (e.g., that pain is uncontrollable, harmful, disabling, etc.) and a lower score indicated more positive health beliefs (e.g., that pain is manageable). The SOPA-B is considered a reliable and valid measure of pain-related health beliefs, with acceptable internal consistency (α = 0.70–0.83), good concurrent validity, and a robust seven-factor structure.^29^ The Cronbach’s alpha coefficient for the SOPA-B scale was 0.77 for the current sample, suggesting good internal consistency.

#### Self-efficacy

The Pain Self-Efficacy Questionnaire (PSEQ)^30^ was used to measure self-efficacy. Participants were asked to rate how confident they felt they could do ten different things despite their menstrual pain on a seven-point Likert scale from 0 ‘not at all confident’ to 6 ‘completely confident’. Statements were summed to produce each participant’s pain self-efficacy score. Scores could range from 0 to 60, with higher scores reflecting stronger self-efficacy beliefs and lower scores reflecting weaker self-efficacy beliefs. The PSEQ has been demonstrated to have excellent internal consistency, test–retest reliability, and construct validity.^31^ The Cronbach’s alpha coefficient for the PSEQ scale was 0.96 for the current sample, suggesting excellent internal consistency.

#### Utilisation of support

How often participants made use of their social support networks was measured using the Utilisation of Support Questionnaire.^32^ Four statements were presented, and participants indicated how often each was true to them on a five-point Likert scale from 1 ‘almost never’ to 5 ‘almost always’. The total score was determined by averaging the responses to the four questions, with a higher mean score reflecting greater utilisation of social support, and a lower mean score indicating poorer utilisation of social support. This approach to measuring social support utilisation has been previously used in the literature.^32^ The Cronbach’s alpha coefficient for the Utilisation of Support Questionnaire was 0.75, suggesting good internal consistency.

#### Satisfaction of support

Participants’ satisfaction with social support was measured using a modified question from a study by Holtzman and colleagues.^33^ ‘Rheumatoid arthritis’ was replaced with ‘menstrual pain’ when participants were asked to indicate who was helpful to them while managing their condition. From the list presented, participants selected as many answers as applied to them. If ‘no one’ was selected, this indicated dissatisfaction with their social support. In contrast, one or more persons selected indicated satisfaction with their social support.

#### Perceived availability of healthcare

Participants were asked whether they knew if there were healthcare appointments available to them. Perceived availability of healthcare was measured by participants either selecting ‘yes’ or ‘no’. This approach has been previously used in the literature.^34^

#### Pain intensity

The Numerical Rating Scale (NRS)^35^ was used to measure pain intensity. Participants indicated how intense their average and most severe menstrual pain was on an 11-point Likert scale from 0 ‘no pain’ to 10 ‘worst pain imaginable’. Higher scores reflected more intense menstrual pain. The NRS is a well-established robust measure of pain intensity,^36^ and has been shown to be a valid and reliable measure of dysmenorrhoea-related pain intensity, specifically.^37,38^

#### Pain interference

Six questions from the Patient-Reported Outcomes Measurement Information System (PROMIS) Item Bank^39^ were used to measure pain interference. Participants indicated how true the statements were to them on a five-point Likert scale from 1 ‘not at all’ to 5 ‘very much’, and how often it applied to them on a five-point Likert scale from 1 ‘never’ to 5 ‘always’. Each question was modified to include ‘menstrual’ before ‘pain’ to measure the pain interference from dysmenorrhoea, specifically. An interference score was determined by averaging the responses to the six questions. A higher mean score indicated greater interference in an individual’s daily life due to their menstrual pain. PROMIS has been found to be a clinically valid measure across several different chronic conditions.^40^ The Cronbach’s alpha coefficient for the PROMIS scale in the current study was 0.94, suggesting excellent internal consistency.

#### Help-seeking behaviour

**Figure 1.**
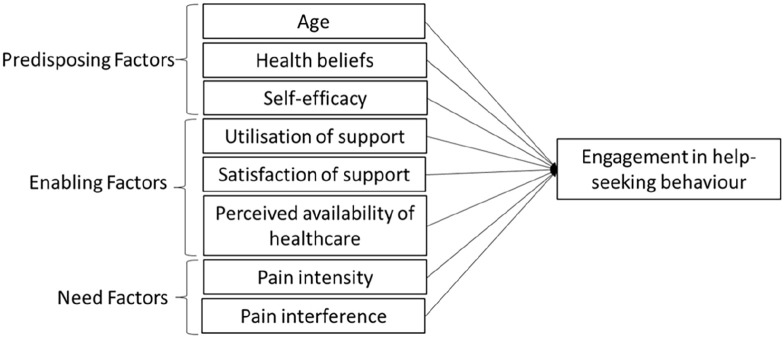
BMHSU predictor variables of engaging in help-seeking behaviour.

The outcome variable of help-seeking behaviour was operationalised as at least one visit to a healthcare professional for menstrual pain.^41^ A healthcare professional was defined as a person who has been educated to provide a healthcare service to a patient such as a general practitioner (GP) or gynaecologist.^42^ As shown in Figure 1, it was hypothesised that the factors described above would predict engagement in help-seeking behaviour.

### Procedure

The General University Ethics Panel of The University of Stirling approved this study prior to its implementation (Ref: GUEP-2023-12913-8944). Data collection took place between November 2022 and December 2023. The survey (see Supplemental material File 2) was pilot tested with two individuals who met the eligibility criteria and then circulated via social media (i.e., Twitter, Facebook, and Instagram) and relevant mailing lists. The purpose of the study was briefed to participants before they electronically gave their informed consent. In their own time, participants completed the survey through the Jisc (2023) online survey platform. A debriefing form was displayed on their screen following the completion of the study. This included a link to the NHS website if participants had further queries or felt distressed due to the personal nature of some questions. Participants who were in their first or second year of study at the University of Stirling and taking a psychology module received a token for their participation.

Given the limitations of the cross-sectional design and opportunity sampling approach, several efforts were taken to minimise additional sources of bias. These included using diverse recruitment channels to reach as broad a sample of the target population as possible, assuring participants of their anonymity to avoid socially desirable responses, avoiding leading questions that might influence responses, and ensuring the survey was accessible on various devices (desktops, tablets, and smartphones) so technical limitations did not prevent participation.

### Statistical analyses

All data were anonymised and stored on a university-affiliated password-protected OneDrive account. Data were analysed using Jamovi v2.2.5.^43^ Listwise deletion was used to remove participants that had missing data. This was to ensure subsequent data analyses produced unbiased estimates of variance, means, and regression weights.^44^ For the demographics and menstrual pain characteristics, descriptive statistics were calculated. Pearson’s correlation coefficients [*r*] and point-biserial correlation coefficients [*r*_pb_] were used to investigate the relationships between continuous, and continuous and binary predictor variables, respectively. Binomial logistic regression was used due to the binary nature of the dependent variable (i.e., engaging in help-seeking behaviour or not). Three blocks were created to signify predisposing, enabling, and need factors as the predictor variables in the BMHSU. Age, health beliefs, and self-efficacy were entered into block one. Support utilisation, support satisfaction, and perceived appointment availability were entered into block two. Finally, pain intensity and pain interference were added to the third block. Dummy variables were used for inputting ‘satisfaction with support’ and ‘perceived availability of healthcare appointments’ as either ‘yes = 1’ or ‘no = 0’ categories.^45^ This analysis aimed to explore the impact of the predictor variables (i.e., predisposing, enabling, and need factors) on help-seeking behaviour. Classification accuracy was used to determine whether the BMHSU was a good model for predicting help-seeking behaviour for dysmenorrhoea, whereby a percentage result above chance indicated that the model fit was sufficient, with a higher percentage indicating better fit.

## Results

### Sample characteristics

A sample of 453 individuals who had reached menarche and were premenopausal participated in the online survey. Of these, 14 cases were removed from the dataset: one was under the age of 16, three did not complete the survey in its entirety, and ten did not experience menstrual pain. This left a sample of 439 participants for analysis. The ages of participants ranged from 16 to 58 with a mean age of 23.7 years (SD = 7.52). Most participants were students (53.76%) or worked part-time (34.4%) or full-time (23.46). The remaining were either unemployed, self-employed, volunteers, or retired (all ~1%).

### Menstrual pain characteristics

For most of the sample (*n* = 286, 65.1%), menstrual pain occurred with every period. Pain occurring in the abdominal region was reported by almost the entire sample (*n* = 430, 98%). For an average period, the mean menstrual pain score was 5.87 (SD = 1.9) and at its most severe, the mean menstrual pain score was 7.83 (SD = 1.8). Thirty-eight participants (8.7%) reported having one or more diagnosed conditions that affected their menstrual pain: 19 (4.3%) had endometriosis; 19 (4.3%) had polycystic ovary syndrome; six (1.4%) had adenomyosis; and one participant each (<1%) reported fibromyalgia, fibroids, pelvic inflammatory disorder, or premenstrual dysphoric disorder. Further details on menstrual pain characteristics are presented in Table 1. In addition to those listed on the questionnaire, other pain sites reported in a free-text box included the pelvic area, breasts, anus, vagina, legs, knees, feet, and neck/shoulders (all ~1%).

**Table 1.** Menstrual pain characteristics.

Characteristic	No.	%
Dysmenorrhoea classification		
No confirmed diagnosis of a gynaecological disorder	401	91.3
Confirmed diagnosis of a gynaecological disorder	38	8.7
Frequency of pain		
Every period	286	65.1
Most periods	106	24.1
Some periods	34	7.7
Few periods	13	3.0
Pain site		
Abdominal region	430	98.0
Lower back	264	60.1
Groin	144	32.8
Thighs	84	19.1
Other	22	5.0

*Note*: The sum of the columns may exceed 100% as categories were not mutually exclusive.

### Sources of healthcare guidance

Most of the sample (66.7%) reported that they had visited a healthcare professional regarding their menstrual pain. Of those, the mean satisfaction score of the healthcare visit was 5.08 (SD = 2.51, interquartile range = 4.0, range = 1–10). The type of healthcare professional most frequently visited was a GP (86%). In addition to those listed on the survey, ~1% of the sample visited a sexual health clinician regarding their menstrual pain and <1% visited a doctor of traditional Chinese medicine. The most used menstrual health information source was speaking to someone they knew (43.96%) or the NHS website (41.69%). Only a small proportion of the sample (13.5%) reported they knew what dysmenorrhoea was before completing the survey. As well as those listed on the survey, other health information sources used to help with menstrual pain were social media and internet searches (both ~3%), and mobile applications (~2%). A full breakdown of sources of healthcare guidance is provided in Table 2.

**Table 2.** Sources of healthcare guidance.

Source of healthcare guidance	No.	%
Visited a healthcare professional for menstrual pain		
Yes	293	66.7
No	146	33.3
Type of healthcare professional visited		
GP	252	86.0
Gynaecologist	86	29.4
Nurse	65	22.2
Pharmacist	31	10.6
Emergency doctor	22	7.5
Physician	7	2.4
Student health services	3	1.0
Other	9	3.1
Type of menstrual health information source used		
Speaking to someone they know	193	44.0
NHS website	183	41.7
Healthcare professional(s)	99	22.6
Blogs	67	15.3
Student support services	10	2.3
Other	81	18.5
None	102	23.2

*Note*: The sum of the columns may exceed 100% as categories were not mutually exclusive.

### Inferential analyses

Descriptive statistics for predictor and outcome variables are displayed in Table 3, and correlation coefficients are provided in Table 4. No strong relationships were found (*r*/*r*_pb_ < 0.4). Variance inflation factor measures were used to check whether the data met the assumption of collinearity. The results indicated that multicollinearity was not a concern for all predictor values (VIF < 2, Tolerance > 0.8).

**Table 3.** Descriptive statistics for each variable.

Variable	*n*	*M* (SD)	No. (%)
1. Age	436	23.7 (7.5)	
2. Health beliefs	427	2.1 (0.5)	
3. Self-efficacy	435	34.3 (14.1)	
4. Support utilisation	438	2.9 (0.8)	
5. Support satisfaction	439		403 (91.8)
6. Appointment availability	435		169 (38.8)
7. Pain intensity	439	7.8 (1.8)	
8. Pain interference	437	3.2 (0.9)	
9. Help-seeking behaviour	439		293 (66.7)

*Note: n* represents the number of participants of the total sample (*N* = 439) who provided complete data for each variable. Variables 5, 6, and 9 were coded as binary variables, whereby 0 = unsatisfied with support, 1 = satisfied with support; 0 = no perceived appointment availability, 1 = perceived appointment availability; and 0 = has not sought help for dysmenorrhoea, 1 = has sought help for dysmenorrhoea, respectively.

**Table 4.** Descriptive statistics and correlations between predictor variables.

Variable	1	2	3	4	5	6	7	8	9
1. Age	–								
2. Health beliefs	−0.23***	–							
3. Self-efficacy	0.02	−0.26***	–						
4. Support utilisation	−0.12**	−0.13**	0.05	–					
5. Support satisfaction	−0.01	−0.14**	0.00	0.33***	–				
6. Appointment availability	0.04	−0.03	−0.03	0.11*	0.12*	–			
7. Pain intensity	−0.03	0.12*	−0.36***	−0.08	0.06	0.01	–		
8. Pain interference	0.03	0.01	−0.07	−0.08	−0.01	−0.04	0.05	–	
9. Help-seeking behaviour	0.08	−0.05	−0.04	0.08	0.14**	0.24***	0.05	0.1*	–

*Note*: – signifies a negative correlation between two variables.

* *p* < .05. **p < .01. ***p < .001.

Binomial logistic regression was used to ascertain the effects of age, health beliefs, self-efficacy, social support utilisation and satisfaction, perceived appointment availability, and pain intensity and interference on the likelihood of engaging in help-seeking behaviour (i.e., having visited a healthcare professional regarding menstrual pain). Regression model coefficients are displayed in Table 5, and model fit measures are displayed in Table 6. The overall model was a better fit on the data than a null model (χ^2^ = 42.94, df = 8, *p* < .001), with a McFadden’s pseudo-R-square value of 0.08. Thus, this model accounted for 8% of the variance in help-seeking behaviour.^46^

**Table 5.** Regression model coefficients.

Predictor	Estimate	SE	*Z*	*p*	OR	95% CI	
						Lower	Upper
Intercept	1.897	1.236	1.534	.125	6.657	0.590	75.065
Age	−0.022	0.016	−1.359	.174	0.979	0.949	1.010
Health beliefs	0.193	0.253	0.765	.444	1.213	0.739	1.992
Self-efficacy	0.006	0.009	0.642	.521	1.006	0.989	1.023
Support utilisation	−0.131	0.147	−0.893	.372	0.877	0.658	1.169
Support satisfaction	−0.696	0.394	−1.767	.077	0.499	0.230	1.079
Appointment availability	−1.118	0.238	−4.694	<.001	0.327	0.205	0.521
Pain intensity	−0.044	0.064	−0.687	.492	0.957	0.843	1.085
Pain interference	−0.295	0.118	−2.502	.012	0.745	0.591	0.938

*Note*: Estimates represent the log odds of ‘Visited healthcare professional? = No’ vs. ‘Visited healthcare professional? = Yes’.

CI: confidence interval; SE: standard error.

**Table 6.** Model fit measures.

Model	Deviance	AIC	BIC	*R* ^2^_McF_	Overall model test		
					χ^2^	df	*p*
1	550	558	574	0.009	4.91	3	.179
2	541	555	583	0.025	13.97	6	.030
3	512	530	566	0.077	42.94	8	<.001

*Note*: AIC: Akaike Information Criterion; BIC: Bayesian Information Criterion; df: degrees of freedom; OR: odds ratio; *R*^2^_McF_: McFadden’s pseudo-R-square.

Perceived appointment availability was a negative and significant predictor of help-seeking behaviour (β = −1.12, SE = 0.24, *p* < .001). This was a dummy variable comparing perceived appointment availability (coded 1 on the variable) with no appointments perceived to be available (coded 0 on the variable). The negative coefficient indicates that individuals who perceived appointments to be available were less likely to have visited a healthcare professional for their menstrual pain. Pain interference was also a negative and significant predictor of help-seeking behaviour (β = −0.29, SE = 0.12, *p* = .01). The odds ratio (OR) indicated that for every unit increase in severity of pain interference, the odds of visiting a healthcare professional for menstrual pain changed by a factor of 0.75, making it less likely they would seek help, indicating that those who experienced greater interference in daily life due to menstrual pain were least likely to visit a healthcare professional.

Prediction measures were calculated to ascertain the adequacy of the BMHSU in predicting help-seeking behaviour. Of the 293 cases that engaged in help-seeking behaviour, 92.8% were correctly predicted by the model. Of the 146 cases that did not engage in help-seeking behaviour, 20.7% were correctly predicted by the model. Overall, the classification accuracy based on the model was 69%, thus indicating the BMHSU is a reasonably good fit for predicting help-seeking behaviour in those with dysmenorrhoea.

## Discussion

To our knowledge, this study is the first to apply the BMHSU to help-seeking behaviour for menstrual pain. Our analysis revealed that the BMHSU had an overall 69% classification accuracy in predicting help-seeking behaviour, indicating reasonably good model fit; however, the model only accounted for 8% of the variance in help-seeking behaviour in this sample. Pain interference and appointment availability were significant predictors of the variance in help-seeking behaviour, such that those who reported greater pain interference and those who perceived greater availability of healthcare appointments were less likely to have visited a healthcare professional for their menstrual pain.

We anticipated that greater menstrual pain interference would be associated with an increased likelihood of past help-seeking behaviour^23^; however, we found that higher menstrual pain interference was associated with a decreased likelihood of an individual having engaged in help-seeking behaviour in this sample. Wakefield and colleagues^47^ found that adolescents with chronic pain that interfered with their daily lives concealed their pain, which may be consistent with the current findings. Individuals in our study whose menstrual pain interferes with their daily lives may not wish to visit a healthcare professional and conceal their symptoms instead. This could be due to the commonly held belief that pain interference during menstruation is ‘normal’.^11,20^ Lengthy delays between symptom onset and diagnosis have been documented in those with secondary dysmenorrhea^48^; though menstrual pain interfered with participants’ daily lives and reduced their quality-of-life, there was a reluctance to visit a healthcare professional. This may be due to individuals’ inability to distinguish between ‘normal’ and ‘abnormal’ menstruation pain. Ultimately, this could explain why pain interference negatively predicted help-seeking behaviour in our sample; individuals may conceal the effects of pain on their lives due to beliefs that this is a normal part of menstruation that may not warrant medical attention.

Perceived healthcare appointment availability was negatively associated with past help-seeking behaviour in this sample; that is, individuals who visited a healthcare professional regarding menstrual pain reported lower perceived appointment availability. This suggests that those who made the decision to engage with health-services faced barriers when trying to access healthcare support.^49^ This may be exacerbated by the recent effects of the COVID-19 pandemic overwhelming the NHS.^50^ Since the pandemic began, primary care utilisation in the UK declined while emergency appointments and non-urgent NHS111 calls increased.^51^ Consequently, those who had previously visited a healthcare professional for their menstrual pain may believe there would not be capacity for them now. Alternatively, they may not wish to utilise limited healthcare resources to discuss menstrual pain despite its potential functional impact. Normalisation of women’s pain is a widespread societal problem that many individuals who experience menstrual pain internalise,^52^ leading them to believe their pain is not worthy of care.^53^ This can reduce the likelihood of help-seeking for even debilitating menstrual symptoms. This could explain why a perceived lack of appointment availability predicted past help-seeking behaviour in our sample; those who have not sought medical help for severe menstrual pain may not realise the potential barriers and overestimate the availability of appointments. Further research is needed to better understand the nature of this relationship.

### Implications

Participants reported moderate-to-severe menstrual pain, with most participants experiencing pain with every period. For guidance on how to cope with menstrual pain, a variety of sources were used; the most common sources were someone they knew, the NHS website, healthcare professionals, and blogs. Although the NHS website and healthcare professionals are the most credible sources,^54^ they were utilised by less than half of the sample. Talking to someone they knew and reading blogs were utilised almost as frequently as the NHS website and speaking to healthcare professionals, respectively. This suggests that a more informal sharing of personal experiences is desired amongst those with dysmenorrhoea. Therefore, narrative-based interventions may be efficacious in encouraging individuals to engage in help-seeking behaviour.^55^ Information booklets containing personal experiences of individuals with dysmenorrhoea can provide positive role models to encourage visiting a healthcare professional if the pain interferes with daily functioning. In this way, valid and reliable information can be made available to the public in a more relatable and accessible format.

Most participants visited a healthcare professional for their menstrual pain, most commonly their GP. This contrasts with previous findings where most individuals did not engage in help-seeking behaviour for their menstrual pain.^19^ However, this study was conducted with UK residents who receive free universal healthcare from the NHS. Finances are a substantial barrier to engaging in help-seeking behaviour in countries outside of the UK with no equivalent free healthcare.^56^ Therefore, it was assumed in this study, removal of financial costs acts as a facilitator of help-seeking behaviour for those with dysmenorrhoea. However, people may be reluctant to visit a healthcare professional due to menstruation-based stigma and a lack of associated menstrual health education and menstrual health literacy.^57^ For those who visited a healthcare professional for their menstrual pain, most reported dissatisfaction with their experience. This is consistent with recent qualitative research,^19,20,58^ which shows people often feel dismissed by healthcare professionals when seeking help for menstrual pain. Healthcare professionals may not be well equipped to provide care for people with dysmenorrhoea due to limited focus on women’s health within medical education.^59^ This has critical implications for the health and well-being of individuals with dysmenorrhoea, as well as those whose pain may be attributable to undiagnosed gynaecological conditions such as endometriosis or polycystic ovary syndrome. More comprehensive education on menstrual and gynaecological health for medical professionals, particularly GPs as the primary point of contact, may improve healthcare experiences for people with menstrual pain.

In our sample, those with more substantial pain interference were also the least likely to have sought healthcare for dysmenorrhoea. Health education interventions may improve engagement with appropriate help-seeking behaviour for those experiencing dysmenorrhoea. Before completing our study, only 13.5% of participants knew what the term ‘dysmenorrhoea’ meant, suggesting a potential lack of menstrual health literacy within the sample. Poor menstrual health literacy may mean that most people may not realise that dysmenorrhoea is a valid condition for which to seek help. This assertion is also supported by the fact that one-quarter of this sample had not accessed any form of menstrual health information despite experiencing pain. This is in line with previous findings that those with poor health literacy did not seek medical help for their menstruation pain.^12^ Therefore, evidence-based menstrual health education should be promoted in schools and the community. This can give people the opportunity to acquire knowledge about the difference between typical and atypical menstrual symptoms and when to engage in help-seeking behaviour. Furthermore, increasing menstrual health literacy can help to reduce societal- and self-stigma associated with menstruation^60^ and promote appropriate help-seeking and effective pain self-management,^20^ resulting in better health outcomes.

### Limitations and suggestions for future research

In our analyses, age, health beliefs, self-efficacy, support utilisation, and support satisfaction did not predict help-seeking behaviour. However, limitations of this study must be considered when interpreting these findings. First, measurement issues should be considered. Although the survey was pilot tested with two individuals prior to dissemination, this was less than 5% of the target sample. This may have limited the usefulness of the pilot for refining the survey. Our help-seeking outcome variable was operationalised as having ever attended to a healthcare professional for menstrual pain, with a binary response option of ‘yes’ or ‘no’. A more sensitive measure of help-seeking behaviour may have yielded more useful results. Additionally, no time frame was defined for this item in the survey, which may further limit the utility of this measure. Furthermore, the SOPA-B was used to measure health beliefs. The brief version was used to reduce the overall length of the questionnaire, and it had been claimed to be comparable to the full Survey of Pain Attitudes (SOPA) scale.^29^ However, research by Jensen and colleagues^61^ found that SOPA scores did not predict the number of visits to a physician in chronic pain patients. Furthermore, Tait and Chibnall^29^ additionally concluded that the SOPA-B had not yet been supported to be as reliable or valid as the long version. However, a trade-off was made due to concerns that a lengthy questionnaire may lower the participation rate due to the response burden. Future research should consider alternative measures of health-related beliefs to enable better understanding of their role in help-seeking behaviour for dysmenorrhoea.

Second, methodological limitations may have impacted the current findings. This study used a cross-sectional observational design; therefore, causal inferences cannot be drawn, nor is it possible to determine the temporal relation between the predictor variables and help-seeking behaviour. For example, as discussed above, it is possible that those who engaged in help-seeking behaviour faced significant challenges in accessing healthcare support, thus resulting in lower perceived appointment availability, while those who have not attempted to seek healthcare for dysmenorrhoea may overestimate the availability of appointments. Furthermore, it is possible that those who have engaged in help-seeking behaviour received effective pain management support, thus explaining the negative relationship with pain interference. Further research utilising longitudinal designs is needed to better understand the factors associated with help-seeking behaviour for dysmenorrhoea and the temporal relationships between these. In addition, there may have been biases in the recruited sample. Non-representative sampling limits the validity of the conclusions we can draw regarding help-seeking behaviour for dysmenorrhoea. People who experience severe menstrual pain or who have had experience of help-seeking for menstrual pain may have been more likely to take part in a study of this nature, thus reducing the generalisability of our findings. Furthermore, the current dataset can offer limited insight into other key factors of interest, for example, the type of healthcare professional visited. Unsurprisingly, most participants who sought healthcare for dysmenorrhoea did so from a primary care provider (*n* = 252, 86%), while only 86 (29.4%) accessed gynaecology services. Accessing specialised healthcare for gynaecological pain is practically and emotionally challenging for many,^52,58^ which has implications for future help-seeking behaviour. More focused research on the experiences and outcomes of those who have sought and received different kinds of healthcare support is needed. Future research should use probability sampling to obtain diverse clinical and community-based samples of people who menstruate to better understand factors involved in predicting help-seeking behaviour and menstrual health outcomes for this group.

Third, we assumed that finances would act as a facilitator to help-seeking behaviour because participants were residents of the UK where public primary healthcare costs are not payable by patients. However, this study failed to recognise that there are other financial costs to visiting a healthcare professional beyond consultation fees. Typically, most healthcare appointments are scheduled on weekdays between 9 am to 5 pm. This means that individuals who work full-time may suffer financial costs to visit a healthcare professional due to lost time from work.^62^ There are also costs associated with transport to and from the location of the appointment and arranging for childcare. Furthermore, if a GP prescribes medication to help with an individual’s symptoms of dysmenorrhoea, there are often charges for prescriptions in England.^63^ Additionally, not all of those who reside in the UK may be considered ‘ordinarily resident’. If an individual does not have ordinarily resident status, they do not qualify for free healthcare from the NHS.^64^ Therefore, it is not possible to claim that finances would act as a facilitator to help-seeking behaviour. In reality, financial costs are likely to be a significant barrier to visiting a healthcare professional for menstrual pain. Future research should consider the role of financial cost in help-seeking behaviour for this group.

Finally, we found that the BMHSU had 69% overall classification accuracy in predicting help-seeking behaviour, which suggests that the model is a reasonably good fit. However, the eight predictor variables only accounted for 8% of the variance observed. This suggests that refinement of the model is required. Future research should investigate whether a different framework could provide better insight into help-seeking behaviour for dysmenorrhoea. For example, the HBM^16^ is used to predict and explain why individuals engage in proactive health behaviours such as visiting a healthcare professional based on several constructs including self-efficacy, perceived severity of the condition, and perceived barriers. The predictor variables we investigated for the BMHSU could instead be mapped onto the HBM to examine which model is a better fit for predicting help-seeking behaviour in a population with dysmenorrhoea.

## Conclusions

Limitations notwithstanding, this study provides preliminary insight into the understudied topic of help-seeking for menstrual pain. Although our analysis suggests reasonably good model fit, the BMHSU does not appear to be a particularly robust model for predicting help-seeking behaviour in those with dysmenorrhoea in the UK. Findings suggest that high pain interference and perceived appointment availability are associated with past non-utilisation of healthcare services for dysmenorrhoea. This may be attributable to poor menstrual health literacy stemming from a lack of menstrual health education and societal attitudes towards women’s pain. Those who did visit a healthcare professional for menstrual pain were mostly dissatisfied with the care provided. Improved medical education on women’s pain and the unconscious biases that affect their care is needed to ensure healthcare professionals have the knowledge and skills needed to diagnose, treat, and refer appropriately. Further research is required to determine the utility of the BMHSU and other theoretical models of health behaviour for predicting help-seeking behaviour in dysmenorrhoea, in order to establish which modifiable factors influence engagement with and experiences of health services for menstrual pain. This would enable future development of public health interventions to promote appropriate help-seeking behaviour and facilitate adequate clinical and self-management of menstrual pain to optimise menstrual health outcomes.

## Supplemental Material

sj-docx-1-whe-10.1177_17455057241273588 – Supplemental material for Help-seeking behaviour in dysmenorrhoea: A cross-sectional exploration using the Behavioural Model of Health Services Use Supplemental material, sj-docx-1-whe-10.1177_17455057241273588 for Help-seeking behaviour in dysmenorrhoea: A cross-sectional exploration using the Behavioural Model of Health Services Use by Sophie C Matheson and Hannah Durand in Women’s Health

sj-docx-2-whe-10.1177_17455057241273588 – Supplemental material for Help-seeking behaviour in dysmenorrhoea: A cross-sectional exploration using the Behavioural Model of Health Services Use Supplemental material, sj-docx-2-whe-10.1177_17455057241273588 for Help-seeking behaviour in dysmenorrhoea: A cross-sectional exploration using the Behavioural Model of Health Services Use by Sophie C Matheson and Hannah Durand in Women’s Health

## Declarations

**Ethics approval and consent to participate:** This study was approved by the General University Ethics Panel of the University of Stirling in January 2023 (Ref: GUEP-2023-12913-8944). Written informed consent was obtained from all participants prior to study participation. In Scotland, individuals aged 16 years and over are presumed to be capable of giving consent on their own behalf to participate in research^27^; therefore, parental consent was not obtained. All procedures were conducted in accordance with the ethical standards of the institutional research committee and with the 1964 Helsinki declaration and its later amendments or comparable ethical standards.

**Consent for publication:** None.

**Availability of data and materials:** Participants did not consent to have their data shared publicly. An anonymised dataset will be made available on reasonable request to the corresponding author.
