# Supplementary material for: Help-seeking behaviour in dysmenorrhoea: A cross-sectional exploration using the Behavioural Model of Health Services Use
Source: Womens Health (Lond). 2024 Aug 20;20:17455057241273588. doi: 10.1177/17455057241273588 (PMC11339737; doi:10.1177/17455057241273588)
Supplement: sj-docx-2-whe-10.1177_17455057241273588 – Supplemental material for Help-seeking behaviour in dysmenorrhoea: A cross-sectional exploration using the Behavioural Model of Health Services Use [file sj-docx-2-whe-10.1177_17455057241273588.docx]

**Supplementary File 2 – Survey Questions**

**What gender do you identify as?**

- Male
- Female
- non-binary
- Other
- Prefer not to say

**What is your age?**

[___]

**Are you currently living in the UK?**

- Yes
- No

**How would you describe your employment status?**

- Full time
- Part time
- Unemployed
- Student
- Other

**Do you experience menstrual pain?**

- Yes
- No
- Prefer not to say

**Do you have a diagnosed underlying condition that affects menstrual pain? (e.g., endometriosis, pelvic inflammatory disease, polycystic ovary syndrome, etc.)**

- Yes – please specify
- No
- Prefer not to say

*Health beliefs: Survey of pain attitudes – brief questionnaire (Trait & Chibnall, 1997)*

Indicate how true each statement is for you. Tick the box of the corresponding number for each statement. Respond to all items.

Use the following scale as a guide:

0 = Very untrue
1 = Somewhat untrue
2 = Neither true nor untrue (or does not apply)
3 = Somewhat true
4 = Very true

1. **There are many times when I can influence the amount of pain I feel**: [0, 1, 2, 3, 4]

2. **I will probably always have to take pain medications**: [0, 1, 2, 3, 4]

3. **When I hurt, I want my family to treat me better**: [0, 1, 2, 3, 4]

4. **I do not expect a medical cure for my pain**: [0, 1, 2, 3, 4]

5. **I have had the most relief from the pain with the use of medications**: [0, 1, 2, 3, 4]

6. **Anxiety increases the pain I feel**: [0, 1, 2, 3, 4]

7. **When I am hurting, people should treat me with care and concern**: [0, 1, 2, 3, 4]

8. **I have given up my search for the complete elimination of my pain through the**

**work of the medical profession**: [0, 1, 2, 3, 4]

9. **It is the responsibility of my loved ones to help me when I feel pain**: [0, 1, 2, 3, 4]

10. **Stress in my life increases my pain**: [0, 1, 2, 3, 4]

11. **Exercise and movement are good for my pain problem**: [0, 1, 2, 3, 4]

12. **Just by concentrating or relaxing, I can ‘take the edge’ off my pain**: [0, 1, 2, 3, 4]

13. **Medicine is one of the best treatments for chronic pain**: [0, 1, 2, 3, 4]

14. **My family needs to learn how to take better care of me when I am in pain**: [0, 1, 2, 3, 4]

15. **Depression increases the pain I feel**: [0, 1, 2, 3, 4]

16. **If I exercise, I could make my pain problem much worse**: [0, 1, 2, 3, 4]

17. **I believe that I can control how much pain I feel by changing my thoughts**: [0, 1, 2, 3, 4]

18. **Often I need more tender loving care than I am now getting when I am in pain**: [0, 1, 2, 3, 4]

19. **Something is wrong with my body which prevents much movement or exercise**: [0, 1, 2, 3, 4]

20. **I have learned to control my pain**: [0, 1, 2, 3, 4]

21. **I trust that the medical profession can cure my pain**: [0, 1, 2, 3, 4]

22. **I know for sure I can learn to manage my pain**: [0, 1, 2, 3, 4]

23. **My pain does not stop me from leading a physically active life**: [0, 1, 2, 3, 4]

24. **My physical pain will never be cured**: [0, 1, 2, 3, 4]

25. **There is a strong connection between my emotions and my pain level**: [0, 1, 2, 3, 4]

26. **I can do nearly everything as well as I could before I had a pain problem**: [0, 1, 2, 3, 4]

27. **If I do not exercise regularly, my pain problem will continue to get worse**: [0, 1, 2, 3, 4]

28. **Exercise can decrease the amount of pain I experience**: [0, 1, 2, 3, 4]

29. **I’m convinced that there is no medical procedure that will help my pain**: [0, 1, 2, 3, 4]

30. **My pain would stop anyone from leading an active life**: [0, 1, 2, 3, 4]

*Self-efficacy: Pain self-efficacy questionnaire (Nicholas, 2007)*

*Please rate how confident you are that you can do the following things at present, despite the pain. To indicate your answer tick one of the numbers on the scale under each item, where 0 = not at all confident and 6 = completely confident.*

1. **I can enjoy things, despite the pain**: [0, 1, 2, 3, 4, 5, 6]

2. **I can do most of the household chores (e.g., tidying-up, washing dishes, etc.), despite the pain**:
[0, 1, 2, 3, 4, 5, 6]

3. **I can socialise with my friends or family members as often as I used to do, despite the pain**:
[0, 1, 2, 3, 4, 5, 6]

4. **I can cope with my pain in most situations**: [0, 1, 2, 3, 4, 5, 6]

5. **I can do some form of work, despite the pain. (“work” includes housework, paid and unpaid work)**: [0, 1, 2, 3, 4, 5, 6]

6. **I can still do many of the things I enjoy doing, such as hobbies or leisure activity, despite pain**:
[0, 1, 2, 3, 4, 5, 6]

7. **I can cope with my pain without medication**: [0, 1, 2, 3, 4, 5, 6]

8.**I can still accomplish most of my goals in life, despite the pain**: [0, 1, 2, 3, 4, 5, 6]

9. **I can live a normal lifestyle, despite the pain**: [0, 1, 2, 3, 4, 5, 6]

10. **I can gradually become more active, despite the pain**: [0, 1, 2, 3, 4, 5, 6]

*Utilisation of support questionnaire (McMurtry et al., 2020)*

**I seek support from others when faced with stress or pain**

[almost never] [seldom] [sometimes] [often] [almost always]

**When I am sick, I find someone to help me with my daily chores**

[almost never] [seldom] [sometimes] [often] [almost always]

**I get invitations to go out and do things with other people**

[almost never] [seldom] [sometimes] [often] [almost always]

**I turn to people for advice or to help solve a problem**

[almost never] [seldom] [sometimes] [often] [almost always]

*Satisfaction with support (Holtzman & Delongis, 2004)*

**Who has been helpful to you in dealing with your menstrual pain – either by talking with you, comforting you, listening to you, giving you advice or giving you practical assistance? Tick all that apply**

- no one
- spouse
- sibling
- child/children
- parent
- parent-in-law
- other relative
- friend
- neighbour
- someone at work
- someone else – please specify

*Perceived availability of relevant health information*

**Are there appointments available for you to speak to a health care professional?**

- Yes
- No
- I don’t know

**What health information sources do you currently use for helping with menstrual pain?**

- NHS website
- Blogs
- Speaking to a health care professional
- Student support from university
- Other – please specify

*Pain intensity: Numerical Rating Scale (Ameade & Mohammed, 2016)*

**Please rate the severity of the menstrual pain you experience from 0 = no pain to 10 = worst possible pain.**

[0, 1, 2, 3, 4, 5, 6, 7, 8, 9, 10]

*Pain Interference: selected PROMIS measures (Cook et al., 2016; Amtman et al., 2010) Short Form 6b*

**How much did pain interfere with your enjoyment of life?**

[not at all] [a little bit] [somewhat] [quite a bit] [very much]

**How much did pain interfere with your ability to concentrate?**

[not at all] [a little bit] [somewhat] [quite a bit] [very much]

**How much did pain interfere with your day-to-day activities?**

[not at all] [a little bit] [somewhat] [quite a bit] [very much]

**How much did pain interfere with your enjoyment if recreational activities?**

[not at all] [a little bit] [somewhat] [quite a bit] [very much]

**How much did pain interfere with doing your tasks away from home (e.g., getting groceries, running errands)?**

[not at all] [a little bit] [somewhat] [quite a bit] [very much]

**How often did pain keep you from socialising with others?**

[Never] [Rarely] [Sometimes] [Often] [Always]

*Utilization of health care services by engaging in help-seeking behaviour*

**Have you spoken to a healthcare professional about your menstrual pain at least once?**

- Yes
- No

*If [no] end questionnaire. If [yes] continue*

**What kind of healthcare professional did you speak to? tick all that apply**

- GP
- Gynaecologist
- Physician
- Pharmacist
- Student health services
- Emergency doctor
- Other – please specify

**Please rate your overall satisfaction with the care you received on a scale from 1-10. With 1 = not satisfied at all, and 10 = totally satisfied.**

[1, 2, 3, 4, 5, 6, 7, 8, 9, 10]
